# A Breakthrough in Understanding the Pathogenesis of Molar Hypomineralisation: The Mineralisation-Poisoning Model

**DOI:** 10.3389/fphys.2021.802833

**Published:** 2021-12-21

**Authors:** Michael J. Hubbard, Jonathan E. Mangum, Vidal A. Perez, Rebecca Williams

**Affiliations:** ^1^Faculty of Medicine Dentistry and Health Sciences, The University of Melbourne, Parkville, VIC, Australia; ^2^Department of Paediatrics, The University of Melbourne, Parkville, VIC, Australia; ^3^Department of Pharmacology & Therapeutics, The University of Melbourne, Parkville, VIC, Australia; ^4^Melbourne Dental School, The University of Melbourne, Parkville, VIC, Australia; ^5^Department of Pediatric Stomatology, Faculty of Health Sciences, University of Talca, Talca, Chile

**Keywords:** global health, paediatric disorders, dental defects, dental caries, medical prevention, developmental biomarkers, serum albumin, biomineralisation

## Abstract

Popularly known as “chalky teeth”, molar hypomineralisation (MH) affects over 1-in-5 children worldwide, triggering massive amounts of suffering from toothache and rapid decay. MH stems from childhood illness and so offers a medical-prevention avenue for improving oral and paediatric health. With a cross-sector translational research and education network (The D3 Group; *thed3group.org*) now highlighting this global health opportunity, aetiological understanding is urgently needed to enable better awareness, management and eventual prevention of MH. Causation and pathogenesis of “chalky enamel spots” (i.e., demarcated opacities, the defining pathology of MH) remain unclear despite 100 years of investigation. However, recent biochemical studies provided a pathomechanistic breakthrough by explaining several hallmarks of chalky opacities for the first time. This article outlines these findings in context of previous understanding and provides a working model for future investigations. The proposed pathomechanism, termed “mineralisation poisoning”, involves localised exposure of immature enamel to serum albumin. Albumin binds to enamel-mineral crystals and blocks their growth, leading to chalky opacities with distinct borders. Being centred on extracellular fluid rather than enamel-forming cells as held by dogma, this localising pathomechanism invokes a new type of connection with childhood illness. These advances open a novel direction for research into pathogenesis and causation of MH, and offer prospects for better clinical management. Future research will require wide-ranging inputs that ideally should be coordinated through a worldwide translational network. We hope this breakthrough will ultimately lead to medical prevention of MH, prompting global health benefits including major reductions in childhood tooth decay.

## Molar Hypomineralisation Is a Global Health Problem Worthy of Prevention

Popularly known as “chalky teeth”, molar hypomineralisation (MH) affects the back teeth (2-year molars, 6-year molars, or both) of 1-in-5 children worldwide, triggering massive amounts of suffering from toothache and rapid decay (Hubbard et al., 2017; Hubbard, 2018, 2020a)^1^ ^,2^. Fortunately this costly condition appears open to medical prevention as, unlike the relatively rare enamel defects termed “amelogenesis imperfecta”, its cause isn’t primarily genetic. Instead, MH seemingly stems from illness earlier in childhood when affected teeth were hardening inside the jaw (Gottlieb, 1920; Hurme, 1949; Suckling, 1989). It follows that aetiological understanding of MH holds remarkable promise as a medical-prevention avenue for improving oral and paediatric health (Hubbard et al., 2017)^3^. However, despite notable exceptions, this enticing opportunity is poorly recognised across the dental/medical/public-healthcare spectrum. Dental awareness of MH has improved significantly over the past 20 years thanks initially to a European-led focus on the commonest variant seen clinically and dubbed “molar-incisor hypomineralisation” (Weerheijm et al., 2001). More recently, a cross-sector translational research and education initiative (The D3 Group)^4^ has brought broader scientific scrutiny on MH alongside other developmental dental defects (D3s) (Hubbard et al., 2017, 2021; Kirkham et al., 2017; Hubbard, 2018, 2020a,b). With these clinico-scientific and translational foundations in place, aetiological understanding is urgently needed to enable better awareness, management and eventual medical prevention of MH.

## Aetiological Understanding Is Fragmentary

Causation and pathogenesis of MH remain unclear despite 100 years of investigation into “chalky enamel”, making it a challenging aetiological problem (Gottlieb, 1920; Suckling et al., 1976; Hubbard et al., 2017, 2021; Fatturi et al., 2019; Silva et al., 2019; Hubbard, 2020a,b). Yet several important advances provide a useful investigative framework to build upon. Foremost, Suckling’s clinicopathological studies in the 1980s clarified that the dental defect involves hypomineralisation, not hypoplasia, of enamel^5^. This knowledge directed attention to the hardening (maturation) stage of enamel development and allied disruption (injury) of the principal enamel-forming cells, termed ameloblasts (Suckling, 1989; Suga, 1989). Secondly, a connection between “chalky enamel spots” (i.e., demarcated opacities, the defining pathology of MH) and systemic infection was proven using an animal model, thereby distinguishing MH from amelogenesis imperfecta and dental fluorosis as originally surmised (Hurme, 1949; Suckling et al., 1983). Thirdly, epidemiology supported a general link with prior illness yet failed to identify specific causes (Suckling and Pearce, 1984; Suckling et al., 1987).

**FIGURE 1:**
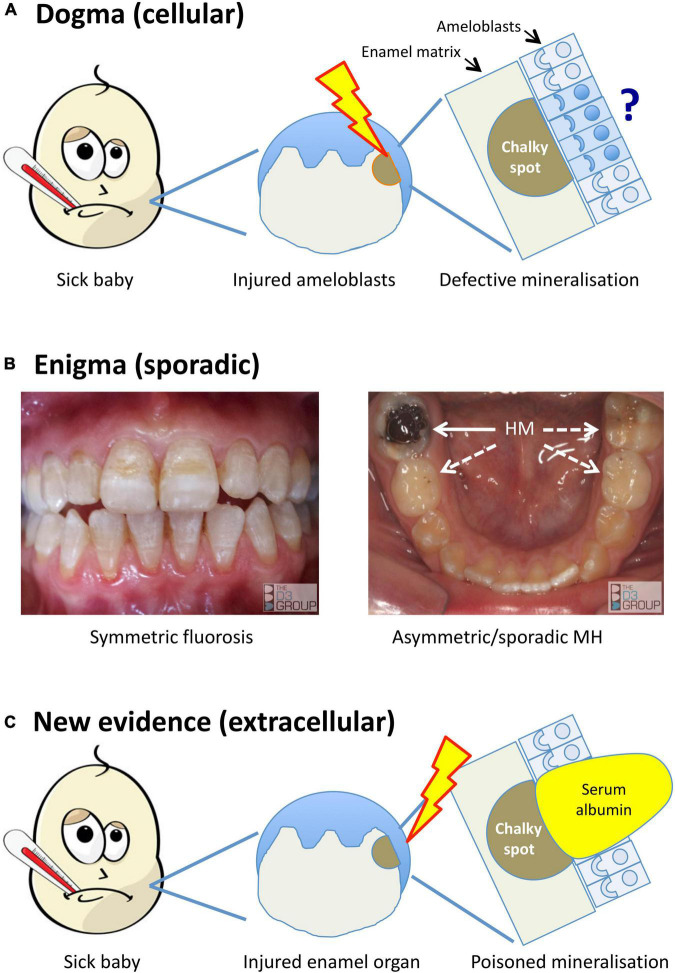
Alternative models for the pathogenesis of MH. Translational portrayal of key pathological concepts presented in the text. **(A)** Current understanding holds that childhood illnesses (*sick baby*) lead to a systemic disturbance that causes cytotoxic injury (*lightening bolt*) to enamel-forming cells (*ameloblasts*). The pathomechanistic relationship between injured ameloblasts (*sad face*) and defective mineralisation of enamel matrix (*chalky spot*) remains unexplained, as does localisation of the injury to some ameloblasts and not others (*question mark*). **(B)** A persisting enigma is that MH presents sporadically, unlike dental fluorosis and other enamel defects (symmetric, chronological) that link logically to systemic disruptions. The *left panel* shows a moderately severe case of fluorosis, with co-linear enamel opacities extending symmetrically across multiple teeth. In contrast, the illustrated MH case (*right panel*) involves a single, severely hypomineralised (HM) 6-year molar (*solid arrow*) – note that despite advanced decay of the affected tooth, its partner on the other side and both 2-year molars appear grossly normal (*broken arrows*). Because MH can involve any number from 1- to all-4 of each molar type (cf. sporadic phenotype), its pathomechanism must contain a localising element. **(C)** New evidence described in the text implicates an extracellular pathomechanism that disrupts enamel-hardening directly (*poisoned mineralisation*) following exposure to serum albumin. Rather than invoking injured ameloblasts primarily, here the injury is envisioned to occur peripherally within the enamel organ (*lightening bolt*), leading to localised exposure to albumin-enriched extracellular fluid (e.g., following vascular leak or haemorrhage). As such, this proposed extracellular pathomechanism has an intrinsic localising element that may account for the clinical characteristics of chalky opacities. Images courtesy of The D3 Group (thed3group.org).

**FIGURE 2:**
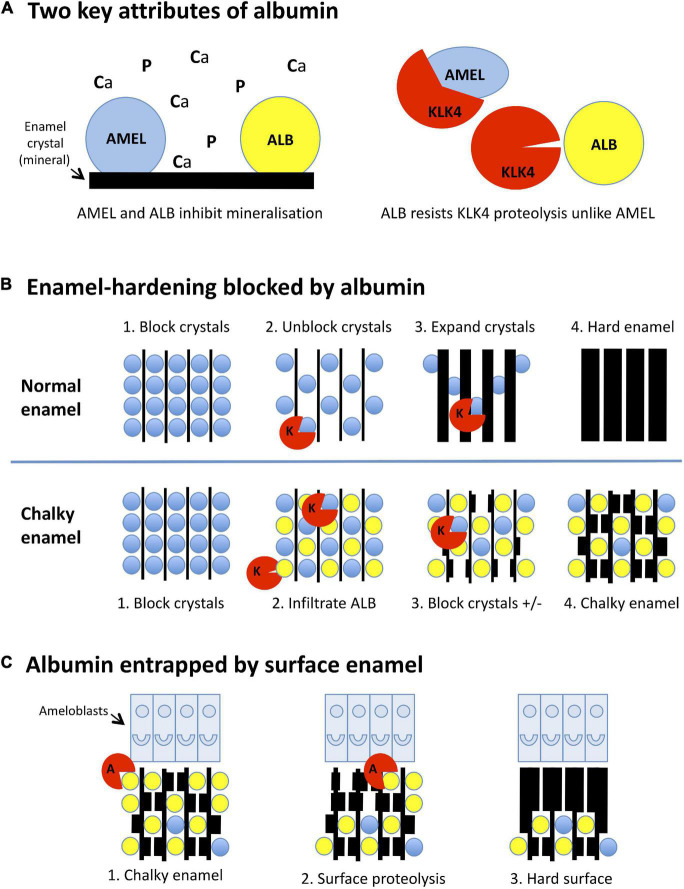
Mineralisation-poisoning model for chalky opacities. Translational depiction of the MP working model described in the text. Colour codings and abbreviations are: amelogenin, *blue*/AMEL; albumin, *yellow*/ALB; kallikrein-4, *red*/KLK4 or K; albuminase, *red*/A. **(A)** Albumin has two key attributes that lead it to disrupt enamel hardening and survive in chalky enamel. (*Left panel*) Like amelogenin, albumin binds tightly to enamel crystals, blocking access of calcium and phosphate ions (*Ca, P*) to the growth surface. Consequently, albumin mimics the crystal-blocking properties of amelogenin. (*Right panel*) In contrast to amelogenin, albumin is resistant to kallikrein-4, the pivotal protease involved in enamel hardening. Albumin can therefore remain adhered to enamel crystals while amelogenin is being degraded. **(B)** Albumin blocks the hardening of enamel. (*Upper panels, 1-4*): (*1*) During the initial (secretory) stage of enamel formation, immature enamel crystals are temporarily blocked from further growth by adherent amelogenin. (*2*) Kallikrein-4 embarks on what will become the complete removal of amelogenin. (*3*) Ensuing ingress of calcium and phosphate enables crystals to expand, eventually producing hard enamel (*4*). In chalky enamel (*lower panels, 1-4*), infiltrated albumin occupies crystal-binding sites left vacant by amelogenin (*1,2*) and uniquely resists degradation by kallikrein-4. (*3*) Crystal expansion is blocked at albumin-bound sites, but proceeds elsewhere following further removal of amelogenin. (*4*) Extension of this process leads to porous chalky enamel containing abundant albumin plus traces of residual amelogenin. **(C)** Subsurface albumin becomes entrapped by hardening of surface enamel. (*1*) Late in the enamel-hardening process, certain albumin-degrading proteases are released by ameloblasts or neighbouring cells. (*2*) Degradation of superficial albumin and amelogenin enables crystal growth at the enamel surface. (*3*) Extension of this process produces an impermeable layer of surface enamel that entraps albumin and other subsurface proteins, culminating macroscopically as an intact chalky opacity.

In the years since, “injured ameloblasts” (Figure 1A) became dogma for MH pathogenesis (Jalevik et al., 2001; Weerheijm et al., 2003; Alaluusua, 2010; Babajko et al., 2017), and a molecular pathomechanism for abnormal hardening (hypomaturation) of enamel was established through investigations of amelogenesis imperfecta and dental fluorosis (reviewed in Mangum et al. (2010a); see Figure 2). Targetted analyses of MH at epidemiological and pathological levels have eliminated some suspected causes [e.g., dioxins (Laisi et al., 2008)] while others remain contentious [e.g., amoxicillin (Laisi et al., 2008; Phipps, 2012)], prompting conclusions that multiple causes and risk factors must be at play (Alaluusua, 2010; Fatturi et al., 2019; Silva et al., 2019). Put another way, if a simple cause-effect relationship existed as for dental fluorosis or tetracycline staining it would likely have been found by now (Mangum et al., 2010a; Hubbard et al., 2017). So, are we missing something, is the injured-ameloblast dogma valid, or are we metaphorically “barking up the wrong tree”?

## Seeking Pathological Clues From Enigma and Unknowns

Accepting that illness in early childhood involves myriad causes and risk factors, an “outside-in” epidemiological approach to MH causation inevitably faces numerous hurdles. The multi-year delay between developmental onset in a sick baby and subsequent eruption of defective teeth adds difficulty (Figure 1). This led us to contemplate a modernised revisit to the pathologic foundations of MH (Suckling, 1989; Suga, 1989). To guide such an “inside-out” approach, a stocktake of key knowns and unknowns was done from a pathogenesis perspective. The broader problem was broken down to five questions, aiming to define pathological hallmarks for MH as follows.

### Q1. Mouth Level – Why Is Molar Hypomineralisation Sporadic?

A long-recognised enigma and still the foremost pathomechanistic clue in our view (Suckling et al., 1976; Suckling and Pearce, 1984; Mangum et al., 2010a; Hubbard et al., 2017), MH is distinguished by its sporadic pattern of attack. That a single patch of enamel on a single molar tooth can be affected in any quarter of the mouth (Figure 1B) contradicts systemic insults playing a deterministic role, as they do in dental fluorosis or tetracycline staining for example. Instead, MH appears to have a localising element embedded in its pathomechanism (Mangum et al., 2010a; Hubbard et al., 2017; Perez et al., 2020).

### Q2. Tooth Level – Why the 6-Year Molars?

Given that any tooth in the baby and adult (primary and permanent) dentitions can be afflicted with demarcated enamel opacities, why are the 6-year (first permanent/adult) molars affected most often? Something besides developmental timing must contribute because other teeth hardening at overlapping ages (i.e., 2-year molars, adult incisors and canines) are affected less frequently (Hurme, 1949; Suckling et al., 1976; Murray and Shaw, 1979)(see text footnote 2).

### Q3. Enamel Opacities – Why Demarcated, Discoloured and Chalky?

Demarcated opacities have readily visible borders with normal enamel, unlike the diffusely bordered opacities of dental fluorosis (Hurme, 1949; Suckling, 1998). What is the reason for this stark difference and the other distinctive clinical features of demarcated opacities (e.g., discolouration, variable “chalkiness”/size/shape/location)?

### Q4. Causation – Disease or Medical Treatment?

Prospects that MH might be caused either by a childhood medical condition, or treatment of that condition, or both in combination, remains a critical stumbling block for epidemiology (Suckling et al., 1987; Alaluusua, 2010; Kuhnisch et al., 2014; Silva et al., 2016). This uncertainty, plus vagueness over the link between age and vulnerability, flags need for alternative aetiological approaches (e.g., animal models).

### Q5. Localising Pathomechanism – Roles for Enamel Cells and Enamel Proteins?

Localised pathomechanistic considerations have focussed on ameloblasts (Figure 1A), leaving many unknowns regarding hallmarks of “chalky enamel”. The need to study involvement of enamel proteins was highlighted by unexplained biophysical findings and prospects for molecular diagnosis (Xie et al., 2009; Farah et al., 2010; Mangum et al., 2010a). Moreover, attractiveness of a biochemical approach was evident from earlier learnings about the inner workings of ameloblasts, as follows.

## A Proteomic Window to the Fragility of Enamel Cells

Pathophysiologically, ameloblasts are widely regarded as fragile cells as exemplified by an unusual sensitivity to fluoride resulting in hypomineralised enamel (Suckling, 1989; Hubbard, 1998a,^2000^). Looking toward prevention, we originally sought to elucidate dental fluorosis mechanistically but later asked whether MH might also be explained by ameloblast fragility. Cell biology fundamentals indicated that enamel cells (i.e., ameloblasts and ancillary cells in the enamel-forming epithelium) would be at risk of calcium-induced injury (cytotoxicity), particularly when handling calcium in bulk during enamel hardening. Yet little was known about the molecular machinery used by enamel cells to deal with bulk calcium safely (Hubbard, 1998a,^2000^). To explore this issue, we developed specialised proteomics procedures for studying many proteins in the tiny amounts of dental tissue available from rodent models (Hubbard, 1998b; Hubbard and Kon, 2002; Mangum et al., 2010b). Enamel cells were found to have equivalent calcium-handling proteins as other cell types, albeit in unusually high amounts (Hubbard, 1995, 1996). Unexpectedly, the protein patterns implied that bulk calcium was transported across the cell using a relatively safe route through organelles, rather than through cytosol as previously believed (Hubbard, 2000; Hubbard et al., 2011). These and later findings indicated that enamel cells are well-adapted to avoid calcium cytotoxicity in normal situations, and also explained their fragility under duress (Hubbard et al., 2011; Nurbaeva et al., 2018). As with other cell types, calcium cytotoxicity provides a unified explanation for how diverse systemic disruptions might lead to common outcomes in injured enamel cells. Although appearing plausible for MH, such cytotoxicity-based mechanisms left the enigma about localised and sporadic injury unresolved (Figures 1A,B). So, with refined proteomics capabilities to hand, attention turned from enamel cells to chalky enamel itself.

## A Categorical Answer About Enamel Proteins in Chalky Enamel

Not knowing the protein composition of chalky enamel was a major shortcoming as noted above. Hypomineralisation defects fall into two categories (hypomaturation, hypocalcification) based on clinical properties and content of amelogenin, the main protein involved in enamel development [reviewed in Mangum et al. (2010a)]. Normally during enamel hardening, amelogenin is removed completely to make way for a dense array of mineral crystals. Hypomaturation defects have abnormally high amounts of residual amelogenin, explaining their failure to harden normally, whereas hypocalcified defects lack amelogenin. Expecting enamel chalkiness to reflect residual amelogenin but also realising other proteins could be involved, we undertook proteomic and amelogenin-specific analyses of hypomineralised 6-year molars. Chalky enamel was found to contain much more protein than normal [3- to 15-fold across different opacities (Mangum et al., 2010a)], seemingly accounting for its mechanical weakness (Xie et al., 2009). Another group made similar findings (Farah et al., 2010). Surprisingly, however, we found only traces of amelogenin, thereby defining chalky opacities as a hypocalcification defect and ruling out a (conventional) hypomaturation mechanism (Mangum et al., 2010a). Realising something other than amelogenin must be responsible for blocking enamel hardening, attention turned to our proteomic evidence that serum albumin was uniquely prevalent in “intact” chalky opacities (i.e., those with a visibly sound, and notionally impermeable, surface).

## A Pathomechanistic Breakthrough for Molar Hypomineralisation

Evidence that albumin (and not amelogenin) was trapped abundantly inside intact opacities but not normal enamel held immediate interest (Mangum et al., 2010a), not least noting earlier arguments for albumin playing a pathomechanistic role in enamel defects (Robinson et al., 1992, 1994, 1996). However, countering clear potential for albumin to *cause* enamel porosity by binding to hydroxyapatite crystals and inhibiting mineralisation as is well understood (Garnett and Dieppe, 1990; Gilman and Hukins, 1994; Yazawa et al., 2000; Yu et al., 2019), several concerns prevailed about experimental artefact – that is, adventitious binding of albumin to *pre-existing* enamel porosity (Couwenhoven et al., 1989; Chen et al., 1995; Wright et al., 1997; Takagi et al., 1998; Robinson, 2014). The origin of “enamel albumin” (i.e., serum albumin located in chalky enamel) was therefore investigated from dental (clinical) and medical (developmental onset) angles, aiming to answer five questions relating to a putative localising pathomechanism (Q5, above), as follows.

### Q5-1. Is Protein Movement in Opacities Restricted by Intact Surface Enamel?

Clinical proteomics had shown that opacities with broken surfaces contained many proteins normally found in saliva whereas albumin alone was abundant in intact opacities (Mangum et al., 2010a). The ensuing premise that infiltration of salivary proteins is blocked by intact (impermeable) surface enamel was supported by our recent evidence that salivary amylase was retained in broken opacities only (Perez et al., 2020). These findings implied that enamel albumin had been acquired developmentally and then become isolated from the oral environment (entrapped) by subsequent hardening of the enamel surface (cf. Figure 2).

### Q5-2. Does Chalky Enamel Contain Foetal Albumin?

Addressing medical onset, we hypothesised that, if chalky enamel in a 6-year molar was actually caused by developmental exposure to albumin during infancy, then the foetal isoform of albumin (alpha-fetoprotein) might be entrapped alongside the postnatal isoform (enamel albumin). Traces of alpha-fetoprotein were indeed found in a majority of chalky opacities and at isoform ratios matching those in neonatal blood. This finding contradicts artefactual exposures at later times – particularly during eruption or extraction – when alpha-fetoprotein is absent (Williams et al., 2020).

### Q5-3. Does Enamel Albumin Show Signs of Ageing?

A second clinical corollary of MH having arisen during infancy is that albumin entrapped for several years in a chalky 6-year molar might be expected to show biochemical signs of ageing. Indeed, enamel albumin was found to exhibit such characteristics (fragmentation, oxidative aggregation) when compared with fresh serum albumin, again supporting a developmental origin (Perez et al., 2020).

### Q5-4. Does Enamel Albumin Correlate With Enamel Chalkiness?

If albumin acts directly as a mineralisation inhibitor (Garnett and Dieppe, 1990; Robinson et al., 1996), a dose-response relationship with clinical hardness seems likely. This mechanistic link was recently upheld by albumin-profiling at gross (chalky vs hard-white opacity) and sub-regional (soft centre vs hard border) levels (Perez et al., 2020).

### Q5-5. Is Albumin Always Absent From Normal Enamel?

Given our finding that normal human enamel lacks albumin (Mangum et al., 2010a), how might contradictory proteomic reports be explained (Farah et al., 2010; Castiblanco et al., 2015; Jagr et al., 2019)? Addressing contamination of surface enamel as a likely contributor, we found that albumin binds rapidly yet disappears along with a variety of other proteins after surface-cleaning. Consequently, normal enamel consistently lacks albumin if surface contamination is avoided (Perez et al., 2020).

Together, these medically- and dentally-directed findings indicated that chalky enamel results from immature enamel being exposed to albumin developmentally, and not at later stages. Now that serum albumin can be reasonably expected to play a direct inhibitory role in enamel (hypo)mineralisation, attention turns to how this local pathomechanistic step might contribute to the broader pathogenesis of MH.

## Working Model: An Extracellular Pathomechanism Involving “Mineralisation Poisoning”

Having answered five key pathomechanistic questions, it seems appropriate to tender a simplified working model for the now-substantiated concept of “mineralisation poisoning” (Perez et al., 2020; Williams et al., 2020). Beneficially, a poisoning metaphor accords with crystallographic terminology and also denotes a process that might be rescued. As depicted in Figure 2, the mineralisation-poisoning (MP) model hypothetically involves two mechanistic elements that together lead to enamel hardening being blocked by entrapped albumin, resulting in chalky opacities. Reductively, three pathologic aspects of MP can be considered, as follows.

### MP-1. Albumin Survives in Hardening Enamel, Unlike Amelogenin

As shown in Figure 2A (*left panel*), albumin mimics amelogenin by binding to immature enamel crystals and blocking entry of mineral ions (Ca, P) to the growth surface. However, unlike amelogenin – which is degraded by the enamel protease, kallikrein-4 (KLK4; *right panel*), and fully removed from maturing enamel – albumin resists such proteolytic attack. Consequently, albumin survives in hardening enamel through a combination of proteolytic stability and tight binding to enamel crystals.

### MP-2. Albumin Blocks Growth of Enamel Crystals Permanently but Incompletely

Normally, enamel hardening is achieved by crystal growth accompanying complete proteolytic removal of amelogenin (Figure 2B, *upper panel*). In chalky enamel (*lower panel*), albumin infiltrates enamel matrix that has been largely depleted of amelogenin, blocking crystal growth permanently at sites where it binds. Subsequent disappearance of residual amelogenin enables localised crystal growth, resulting in porous chalky enamel.

### MP-3. Albumin Becomes Entrapped by Subsequent Formation of Surface Enamel

We posit that, although albumin in subsurface enamel survives attack by kallikrein-4, other proteases that actually can degrade albumin exist at the enamel surface (Figure 2C). Enamel cells might secrete such “albuminases” normally as part of surface hardening. Alternatively, albuminase could be released incidentally by other cells involved in the pathologic event. Either way, removal of superficial albumin would lead to subsurface albumin becoming entrapped under an impermeable opacity surface.

As such, the MP model has two main differentiators from the ameloblast-based dogma (Figure 1). First, the ultimate pathologic event involves extracellular albumin instead of injured ameloblasts (i.e., poisoned mineralisation vs cytotoxicity). Second, the direct link with extracellular fluid (in which albumin is the predominant protein) provides an intuitively simple explanation for the varied characteristics of chalky opacities (cf. Q3, above). It seems plausible that individual variations in chalkiness, size, shape and location might correlate with differences in exposure to albumin. Likewise, the complex anatomy of some opacities might reflect links between albumin seepage and extent of amelogenin removal (i.e., non-uniform permeability of enamel matrix). The distinctive discolouration of chalky enamel also harmonises with albumin being a carrier of small molecules, chromophores included (Fasano et al., 2005). While diverting ultimate attention from ameloblasts, this new thinking preserves the link to childhood illness because extracellular fluid is affected by numerous medical conditions, both quantitatively and qualitatively. We also envision that, by acting as an epithelial barrier, enamel cells might still play a penultimate role in gating access of albumin to the enamel matrix (Hubbard, 2000). Although uncertainties abound, this working model provides a useful foundation for further pathomechanistic enquiry.

## Next Hypothetical Steps – “Fossilised Blood Proteins”

Besides provisionally accounting for several hallmarks of chalky opacities, the MP model might also explain the sporadic nature of MH (cf. Q1 above; Figure 1B) as anticipated (Mangum et al., 2010a). To explore this notion of stochastic localised disruption, it will be necessary to learn where enamel albumin came from and whether serum albumin is the sole disruptor of enamel hardening. The simplest prospect is that enamel albumin derives from blood, either as vascular leak (tissue fluid, oedema) or haemorrhage. It follows that other blood-derived proteins satisfying the survival criteria (Figure 2A) may also be retained in chalky enamel alongside albumin. Hence we now hypothesise that, in translational terms, chalky opacities represent “fossilised blood proteins”. Moreover, given the trace amounts of amelogenin found in chalky enamel (Mangum et al., 2010a), it seems plausible that blood-derived modulators (e.g., inhibitors of kallikrein-4) may affect amelogenin removal. Indeed, supportive data have emerged from ongoing biochemical analyses (Perez et al., 2018)^6^, thereby broadening pathomechanistic interest to blood derivatives in general rather than albumin alone. As such inside-out biochemical understanding expands, epidemiological and animal-based approaches will be needed to test the refined model.

## Translating From Scientific Breakthrough to Cross-Sector Action

Despite its nascent evidence-base, our working model (Figure 2) provides the strongest explanation for the ultimate local step of MH pathogenesis to date – for the first time in a century of enquiry, the topographical and biophysical characteristics of demarcated opacities can be rationalised coherently. Our inside-out approach provides a pathological base in clinical specimens that has identified diagnostic biomarkers for chalky enamel and raises new aetiological questions invoking a range of medical conditions. Future research directed at medical prevention and better clinical management will require wide-ranging inputs (e.g., scientific, medical, dental, epidemiological, pharmacological) embracing various population settings. Thus, a globally-networked translational effort should be encouraged if the enticing prospects for improved oral and paediatric health are to be realised effectively (Hubbard et al., 2017, 2021; Hubbard, 2020a,b).

## Conclusion

The 100-year pathomechanistic question about chalky enamel spots affecting 1-in-5 children worldwide has now met a surprising answer (Hubbard, 2020a; Hubbard et al., 2021). Specifically, a ground-breaking series of biochemical and proteomics investigations has revealed the localised failures in enamel hardening are ultimately associated with developmental exposure to serum albumin – a blood-derived protein that poisons the growth of mineral crystals – not ameloblast injury. These findings offer prospects for better clinical management of MH and open a new direction for research into its broader pathogenesis and causes. We hope this breakthrough will eventually lead to medical prevention of MH, prompting global health benefits including major reductions in childhood tooth decay (see text footnote 3).

## Data Availability Statement

The original contributions presented in the study are included in the article/supplementary material, further inquiries can be directed to the corresponding author.

## Author Contributions

MH conceived, designed and wrote the article and figures based on intellectual content developed collaboratively with JM, VP, and RW. All authors refined and reviewed the final manuscript.

## Conflict of Interest

MH is founder/director of The D3 Group for developmental dental defects (www.thed3group.org), a charitable network. The remaining authors declare that the research was conducted in the absence of any commercial or financial relationships that could be construed as a potential conflict of interest.

## Publisher’s Note

All claims expressed in this article are solely those of the authors and do not necessarily represent those of their affiliated organizations, or those of the publisher, the editors and the reviewers. Any product that may be evaluated in this article, or claim that may be made by its manufacturer, is not guaranteed or endorsed by the publisher.

## Footnotes

1
www.thed3group.org/what-are-chalky-teeth.html

2
www.thed3group.org/prevalence.html

3
www.chalkyteeth.org/prevention.html

4
www.thed3group.org

5
www.thed3group.org/grace-suckling.html

6 Our unpublished data (MH, VP, JM).

## Abbreviations

MH: molar hypomineralisation
D3: developmental dental defect
MP: mineralisation poisoning.
